# The Benefits and Challenges of Patient-based Blood Pressure and Electrocardiogram Measurements at Home

**DOI:** 10.19102/icrm.2019.100905

**Published:** 2019-09-15

**Authors:** Jonathan S. Steinberg

**Affiliations:** ^1^University of Rochester School of Medicine & Dentistry, Rochester, NY, USA; ^2^Hackensack Meridian School of Medicine, Seton Hall University, South Orange, NJ, USA; ^3^SMG Arrhythmia Center, Summit Medical Group, Short Hills, NJ, USA

**Keywords:** Atrial fibrillation, blood pressure monitoring, ECG interpretation, hypertension, stroke


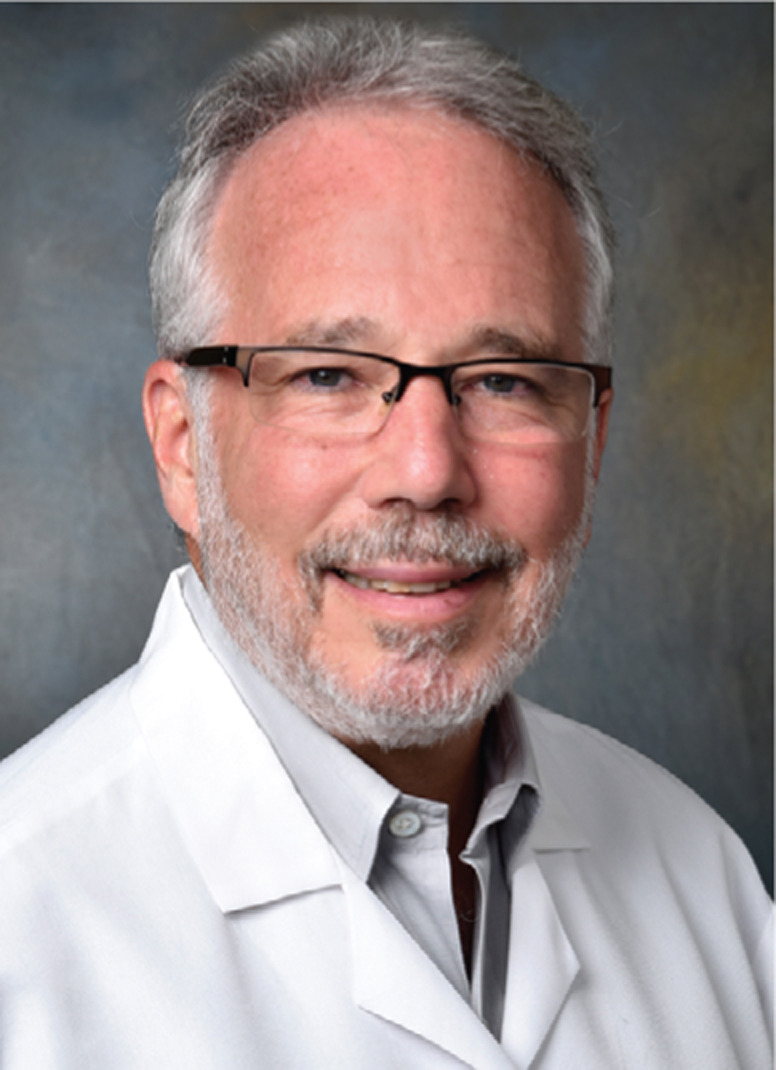


## Introduction

With the United States Food and Drug Administration’s recent clearance for market of several products and technologies, the age of consumer-initiated electrocardiogram (ECG) recordings is without question upon the medical community. While this development offers opportunities, many challenges also still must be successfully navigated. Among others, the electrophysiology community is expected to be on the front lines of this revolution in care.

## About Dr. Steinberg

Professor Jonathan Steinberg, MD is the Director of the SMG Arrhythmia Center at Summit Medical Group, the largest independent medical group in the United States and a Professor of Medicine at the University of Rochester School of Medicine and Dentistry as well as a Core Professor of Cardiology and Internal Medicine at Hackensack Meridian School of Medicine at Seton Hall University. He is a fellow of several professional societies including the American Heart Association, the American College of Cardiology, the American College of Physicians, the European Society of Cardiology, and the Heart Rhythm Society. He is the immediate past President of the International Society of Holter and Noninvasive Electrocardiology.

Dr. Steinberg has participated in virtually all of the seminal randomized clinical trials in electrophysiology and has held executive or leadership positions in many. He is currently the recipient of National Institutes of Health funding for a trial exploring whether atrioventricular junctional ablation improves the response to cardiac resynchronization therapy in patients with atrial fibrillation (AF).

## Interview

***Question:*** What do you as a physician see as the value in measuring both blood pressure (BP) and ECG at home?

***Dr. Steinberg:*** Patients have been measuring their own BP for a very long time and the data obtained in this manner have been useful for clinical care including in the diagnosis of hypertension and the optimization of individual medical care. Patients with hypertension are vulnerable to the development of AF and home BP monitors have historically alerted patients to “irregular” rhythms, but this finding to date has not been corroborated for accuracy with an ECG. Simultaneous ECG recording with BP measurement would be a highly desirable advance and would provide tangible data to the caregiver that could benefit both individual patient prognoses and medical care.

***Question:*** What type(s) of patients do you view as good candidates for at-home ECG measurement? Why?

***Dr. Steinberg:*** At present, ideal patients would be those with a known diagnosis of AF for whom a better characterization of AF burden and patterns will be clinically useful for management. For example, patients who are being titrated on medical therapy or who have undergone catheter ablation often undergo circumscribed ambulatory ECG monitoring, but prolonged at-home ECG recording may serve as a complementary or alternative to these traditional techniques of arrhythmia detection. Simultaneous BP measurements for hypertensive patients would facilitate the optimization of antihypertensive therapy, which in itself may be antiarrhythmic.

***Question:*** Omron Healthcare (Court Lake Forest, IL, USA) recently launched a device, Complete™, that allows for the simultaneous measurement of BP and ECG at home. Do you feel that the usage of such devices in general can help physicians to avoid unneeded, in-person patient visits? Why or why not?

***Dr. Steinberg:*** Yes. Empowering patients to participate in their own care is very valuable. Further, the patient can be given instructions to repetitively capture clinical data that can be provided to the physician to inform treatment decisions. There is no reason that data acquired directly by the patient with such a kind of device cannot be used efficiently and productively such that patient care visits are diminished. This type of partnership is particularly advantageous for patients who have many competing clinical care needs, who are elderly or infirm, or who are managed in health care systems that seek to optimize clinical care efficiency.

***Question:*** In general, as at-home self-monitoring becomes more commonplace in health care, what do you see as potential challenges that the physician community may face?

***Dr. Steinberg:*** There are a number of challenges. Many patients will have a low pretest probability, which will place a premium on test specificity. Some guidelines now recommend pulse palpation as one simple and inexpensive method to identify abnormal rhythms such as AF. Other, more expensive tools will need to demonstrate their value before there can be widespread adoption. Among consumer products, the rigorous evaluation of competing technologies deserves careful assessment. The relative advantages of detection by heart rate, tachograms, and ECGs should be formally tested. Even once a strategy is implemented, it is unclear for a time what the optimal frequency and duration of monitoring are.

However, direct empowerment of the patient may generate excess anxiety in some that could counteract certain advantages. Most ECG recordings will also ultimately require physician interpretation, which could overwhelm contemporary systems and practices. Processes and systems will need to be adopted to manage this new data flow and incorporate the collected information into patients’ records. However, the time and effort to do so may tax the models that are currently in place, especially with respect to professional compensation. Some have voiced concerns that downstream cardiac testing will be unnecessarily increased and add expenses to patient care.

Clinical outcome benefits and cost-efficacy will be the final gold standards, but relevant data are scarce at the present time.

***Question:*** How, in your opinion, should general practitioners handle the ECG strips, given that they are not as familiar as cardiologists with them?

***Dr. Steinberg:*** Based on my own experience and published literature, it would be best if primary care physicians partnered with a cardiologist who is comfortable with interpreting these ECG strips (although not all are) or an electrophysiologist.

***Question:*** In general, patients have a fear of stroke and a fear of dementia. How can a home device with both BP measurement and ECG recording functions be used to address these concerns?

***Dr. Steinberg:*** AF, of course, places patients at the risk of both stroke and dementia. So, surveillance ECG recording is being evaluated to determine if this is a cost-effective strategy for the diagnosis of AF beyond the usual office ECG recordings or further ambulatory ECG recordings if patients have symptoms. However, this is still an unsettled question and there is a great deal of ongoing investigation. Even more up in the air is whether or not intervention with anticoagulant therapy can prevent these serious events in a population of patients who have AF detected in this manner, who may differ in terms of their long-term risk from traditionally diagnosed AF patients. Nonetheless, I suspect that patients will pursue this course of action on their own out of the concerns you have raised above until there are more definitive clinical trial data available. The use of artificial intelligence and machine learning has the potential to improve the accuracy of automated interpretations.

***Question:*** What is the process for physicians to get paid for reviewing patient home monitoring results, such as BP and ECG strips? Are there any kinds of CPT codes available in the United States for such payments? To your knowledge, how successful have physicians been in getting the necessary reimbursements?

***Dr. Steinberg:*** It is a challenge to be reimbursed but there is some progress. CPT code 99091 (approved in 2018) states that reimbursement can be requested “for time spent on [the] collection and interpretation of health data that is generated by a patient remotely, digitally stored and transmitted to the provider, at a minimum of 30 minutes of time/month” and will provide 1.63 relative value units ($58.68).

***Question:*** In your opinion, what kind of role should a pharmacist play in helping patients or consumers select the right home devices and/or in helping them to understand the right way(s) of using such devices?

***Dr. Steinberg:*** Pharmacists are on the front lines and often have strong relationships with individual patients as well as experience a great deal of face-to-face contact with them. They can provide advice on monitoring options, tailored to individual patients’ needs, and inform them of current indications and techniques.

***Question:*** What kind of role might a smartphone app play in home monitoring? What kinds of basic features should an app have to be useful, from both the patient and health care professional perspectives?

***Dr. Steinberg:*** The smartphone has in many ways become an extension of a medical office visit. As such and because of its ubiquitous presence, it can be very advantageous to perform on-the-spot sampling of physiologic parameters at any time or place. However, the quality of the data can be highly variable and often collected and analysed using poorly validated algorithms. For now, it is very helpful to be able to view the source material before it has been processed by an app or before an interpretation has been finalized. Some basic features can include heart rate, activity level, a tachogram, and ECG samples.

***Question:*** What is the value of concurrent BP measurement and ECG recording at home? What is the value of patient daily monitoring at home for an extended period of time?

***Dr. Steinberg:*** Assessing for a diagnosis of hypertension or for the optimal treatment of hypertension if a patient is already diagnosed makes regular BP recordings valuable in patients with AF. Serial recordings provide repetitive data samples and increase data integrity.

***Question:*** Do you feel the availability of devices like Complete™ will create a higher level of physician recommendation for at-home monitoring? Why or why not? If not, what do you feel are barriers to adoption/what challenges need to be overcome?

***Dr. Steinberg:*** A combination device like Omron Healthcare’s Complete™ product provides a very efficient and convenient avenue for the simultaneous collection of BP and ECG, and I suspect will be quite popular with caregivers. The concern on the part of physicians will be the data deluge that can follow.

***Question:*** For patients already monitoring BP at home, what additional value do you feel exists for the physician to have access to simultaneously recorded ECG data? How might a physician use this unique information?

***Dr. Steinberg:*** For some patients who monitor BP, a home-based ECG may be clinically valuable, but probably not necessary for every patient. Older patients, those with unexplained symptoms, and those with a high risk of stroke if AF were to be diagnosed may be good candidates for simultaneous ECG recordings.

